# DG-FuseNet: A Dual-Scale Dynamical Gated Fusion Framework for Cross-Domain Fault Diagnosis in Rotating Machinery

**DOI:** 10.3390/s26102968

**Published:** 2026-05-08

**Authors:** Dawei Zhang, Xiaoheng Deng, Yun Liao, Jinsong Gui, Jingjing Zhang

**Affiliations:** 1 School of Electronic Information Engineering, Central South University, Changsha 410083, China or zhangdawei@tangzhihn.com (D.Z.); jsgui2010@csu.edu.cn (J.G.); jingjingzhang@csu.edu.cn (J.Z.); 2 Tangzhi Science & Technology Hunan Development Co., Ltd., Changsha 410007, China; liaoyun@tanzhihn.com

**Keywords:** cross-domain fault diagnosis, dual-scale gated fusion, fast Fourier convolution, rotating machinery fault diagnosis, variable-speed conditions, wavelet convolution

## Abstract

**Highlights:**

**What are the main findings?**

- Strong anti-interference capability: DG-FuseNet has excellent interference suppression ability and can maintain high diagnostic accuracy even in noisy environments. Under different signal-to-noise ratio (SNR) levels (from −10 dB to 2 dB), DG-FuseNet significantly outperforms other models. Especially under extreme noise conditions, the degradation of its performance is much smaller than that of other models.
- Powerful generalization performance: The model demonstrates strong generalization capabilities in complex industrial environments, such as train running components and aero engines, and can adapt to variable operating conditions and failure modes.

**What are the implications of the main findings?**

- High-order interpolation tracking resampling (HOITR): It effectively addresses the difficulty of scale normalization under variable-speed conditions, resampling nonstationary signals in the time domain into an approximately order-stationary angular-domain representation, thereby overcoming the limitations of traditional Fourier transforms.
- Dual-branch feature extraction: By combining the fast Fourier convolution (FFC) branch and the wavelet convolution (WTConvNext) branch, it can effectively extract local and global time–frequency information.
- The dynamic gating fusion module is introduced to adaptively adjust the weight coefficients of the input features, effectively fusing the dual-scale features extracted by fast Fourier convolution and wavelet convolution branches, thereby enhancing the model performance, especially when dealing with complex tasks and multimodal data.

**Abstract:**

To address the challenges of insufficient scale normalization, limited time–frequency localization, and ineffective multi-scale feature extraction in the intelligent fault diagnosis of rotating components under varying operating conditions, we propose a novel convolutional neural network, termed DG-FuseNet. The proposed method was validated on real-world datasets from train vehicle vibration signals and aero-engine systems, achieving diagnostic accuracies of 99.76% and 94.32%, respectively. Compared with eleven advanced intelligent models, DG-FuseNet demonstrated faster convergence, higher diagnostic accuracy, strong robustness against interference, and superior generalization capability. These results indicate that DG-FuseNet outperforms existing approaches in complex industrial scenarios, highlighting its excellent performance and stability.

## 1. Introduction

The advent of intelligent manufacturing and Industry 4.0 underscores the critical role of mechanical rotating components, which serve as indispensable elements in industries such as manufacturing, aerospace, and transportation [1]. Failures of these components not only result in substantial economic losses but also pose serious safety risks. In industrial environments, fault diagnosis is particularly challenging due to complex operating conditions, harsh surroundings, and diverse vibration patterns. Consequently, developing algorithms capable of effectively analyzing vibration signals from rotating machinery is vital for accurate fault detection [2].

In recent years, the integration of machine learning and deep learning has opened new paths for intelligent fault diagnosis [3]. These techniques have achieved significant progress in data-driven intelligent fault diagnosis. These techniques have improved fault classification accuracy, enhanced the efficiency of mechanical systems, strengthened safety monitoring, and reduced maintenance costs [4]. Although these methods have somewhat enhanced fault diagnosis accuracy, challenges remain in processing complex industrial data and generalizing fault mode classification.

Firstly, models that rely exclusively on CNNs are susceptible to information loss. Conventional lightweight strategies, such as unstructured pruning, parameter sharing, and local attention mechanisms, can effectively reduce computational overhead during training; however, they may also unintentionally eliminate critical information. For example, the local attention mechanism, by emphasizing limited portions of the sequence, may fail to capture important global features [5,6]. Moreover, CNN-based fault identification methods primarily extract features from time-domain signals under the assumptions of constant operational speed and abundant training data. Consequently, these approaches often exhibit degraded performance when confronted with limited samples or varying speed conditions. Small datasets restrict the model’s capacity to learn diverse fault features, while speed fluctuations introduce instability and unpredictability in time-domain signals, thereby complicating fault feature extraction. Collectively, these issues reduce generalization ability and impair classification accuracy due to feature discrepancies induced by variations in rotational speed [7,8].

Secondly, many of the aforementioned algorithms fundamentally depend on a single modality of feature input for fault characteristic extraction. Such reliance substantially limits their capacity to capture the full spectrum of multi-scale and multi-dimensional information, thereby constraining their ability to fully exploit the raw signals of rolling bearings. Consequently, their effectiveness in identifying complex fault relationships is significantly restricted. Furthermore, when confronted with intricate defect mechanisms and noise interference, reliance on a single input modality critically weakens the model’s ability to recognize and learn essential features, ultimately resulting in reduced diagnostic accuracy and poor generalization. To advance fault diagnosis in rolling bearings, it is essential to adopt multimodal approaches that leverage the complementary strengths of diverse data sources, thereby enabling more comprehensive and accurate fault detection.

Third, conventional convolution operations in modern deep networks are typically localized and limited in scope, thereby reducing efficiency in capturing long-range dependencies. Recently, new convolutional operators have been introduced to effectively model non-local receptive fields and integrate multi-scale information. In deep convolutional neural networks (CNNs), the receptive field is a fundamental concept, closely associated with the structural characteristics of the human visual system. Most modern architectures employ stacked convolutional kernels with restricted receptive fields. Although this design can expand the receptive field in a linear or exponential manner, it still encounters limitations in context-dependent tasks that require large receptive fields, such as fault diagnosis of rotating machinery. Moreover, achieving cross-scale feature fusion without incurring excessive computational costs remains a significant challenge. To address these issues, researchers have proposed approaches such as deformable convolution and non-local neural networks to broaden the receptive field.

The remainder of this article is organized as follows. Section 2 introduces the theoretical foundations and methodologies of the proposed DG-FuseNet approach. Section 3 details the design process and implementation of DG-FuseNet. Section 4 describes the dataset composition, outlines the experimental framework, and evaluates the model’s performance on benchmark datasets. Finally, Section 5 summarizes the key findings and discusses potential directions for future research.

## 2. Materials and Methods

Time-varying rotational speed introduces two major challenges. The first is spectral blurring: variations in rotational speed cause drifting of fault characteristic frequencies, such as bearing fault frequencies and gear meshing frequencies. When analyzed using the conventional FFT method, this drift results in spectral smearing, thereby hindering accurate feature extraction. The second is order aliasing: nonlinear variations in rotational speed lead to overlapping of order components in the frequency domain, a phenomenon that becomes more severe in higher-order harmonic regions. To address these issues, the High-Order Interpolative Tracking Resampling (HOITR) method has been developed. HOITR is specifically designed to overcome the limitations of traditional Fourier transforms when analyzing complex signals. It first monitors the rotational speed in real time and employs high-precision sensors to capture instantaneous rotational speed information. These data are then utilized to resample the original signal, stabilizing it in the angular domain such that signal variations are synchronized with the rotation angle. Through this transformation, a previously complex, time-varying signal can be represented as a sequence of periodic and stable components in the angular domain, thereby greatly simplifying subsequent order analysis and fault diagnosis [9].

The control systems of the experimental platforms may affect the collected vibration signals. In the MTRG dataset, the traction, braking, and vehicle operation control systems can change the speed and load profiles of the running gear, resulting in time-varying vibration amplitudes and frequency components. In the HIT dataset, the LP/HP motor drive system may introduce speed regulation errors, torque fluctuations, and transient responses during acceleration, while the lubricating and temperature control system may influence the friction state and vibration amplitude of the inter-shaft bearing.

These control-related factors increase intra-class variability and may cause domain shifts between different operating conditions. In the proposed framework, HOITR is used to reduce the influence of rotational speed fluctuation by transforming the signal from the time domain to the angular domain. The FFC branch then extracts global order-related periodic features, whereas the WTConvNext branch captures local transient and multi-scale details. The dynamical gated fusion module further improves robustness by adaptively balancing these two types of features.

To overcome the limitations of local receptive fields, where most existing CNN models employ small convolutional kernels that restrict the ability to capture spatially distant features, current approaches remain insufficient for tasks requiring global context, such as diagnosing rotating components. To address this challenge, T. Chu et al. [10] introduced fast Fourier convolution (FFC) by emphasizing the importance of cross-scale fusion. They observed that feature maps at different layers of CNNs contain information at multiple scales, and cross-scale fusion can effectively exploit this complementary information to enhance the model’s representational capacity. FFC further integrates local and global convolution within a single computational unit by leveraging the properties of the Fourier transform. Specifically, the Fourier transform converts spatial data into the frequency domain, where modifying a frequency domain coefficient can influence the global feature response after inverse transformation, thus providing a theoretical foundation for constructing a global receptive field. By addressing the constraints of local receptive fields and enabling effective cross-scale fusion, FFC significantly improves model performance in defect classification.

Wavelet convolutional neural networks (Wave-CNNs) integrate wavelet transforms with convolution operations to enhance multi-scale feature representation [11]. Wavelet basis functions provide effective time–frequency localization, enabling the decomposition of input signals into high- and low-frequency components during convolution and thereby capturing information across multiple scales, such as edges and textures. A common strategy is to replace conventional convolutional kernels with wavelet functions to achieve sparse representation, while constructing dual-path networks that operate in both the wavelet and spatial domains.

Dynamic neural networks [12] adjust their mapping relationships according to the input, enabling adaptability to new data and operating conditions. This flexibility makes them particularly suitable for tasks involving varying data complexity. In the context of fault diagnosis for rotating machinery, such networks may adapt their structure based on current sampling conditions to effectively process signals represented in different transformation domains, such as the Fourier and wavelet transforms of vibration data. Weight-dynamic networks and gated fusion mechanisms have also been applied in diverse vision tasks, including image dehazing and semantic segmentation [13]. In these applications, the dynamic structure adapts to the input to integrate multiple types of information within the gated fusion block.

## 3. DG-FuseNet Model Description

### 3.1. Model Architecture and Design

The original vibration data collected on-site is first linearly interpolated, while the rotational speed pulse data is frequency-doubled to generate a resampled pulse sequence. After verification, the vibration data is resampled, and the angular-domain sampling resolution is normalized to 400 samples per revolution. This process unifies the diagnostic scale for nonstationary speeds and rotating components operating at different speeds across various industrial scenarios, thereby mitigating the problem of spectral line blurring that occurs when directly applying the Fourier transform to raw time-domain data. Once processed by High-Order Interpolative Tracking Resampling (HOITR), the data is simultaneously fed into both the fast Fourier convolutional branch and the wavelet convolutional branch, enabling split processing that incorporates both local and global scales.

After global and local feature extraction, the features are passed through a dynamic gating mechanism to achieve dual-scale feature fusion. This process effectively integrates information from different levels and scopes, thereby enhancing the accuracy of fault diagnosis. Finally, classification is performed on the fused features to generate diagnostic results for rotating components, enabling users to promptly assess equipment operating conditions and undertake necessary maintenance actions.

**Figure 1:**
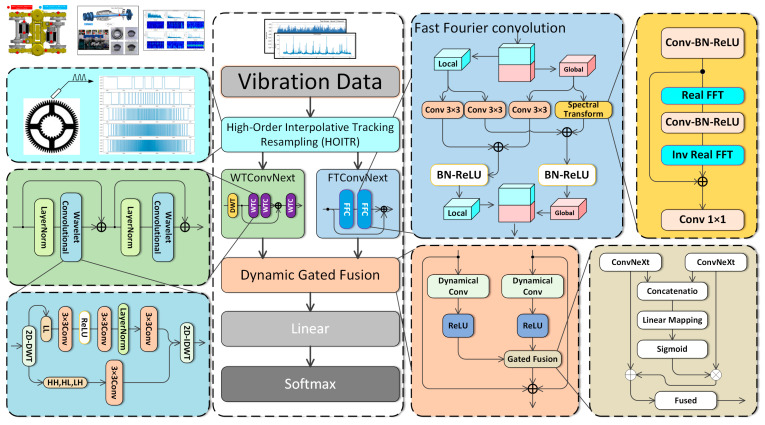
Overall architecture diagram of the DG-FuseNet model.

Overall, as illustrated in Figure 1, the proposed method first applies high-order tracking resampling to the raw industrial vibration data, effectively addressing scale normalization issues caused by velocity fluctuations. The resampled data is then processed through two parallel branches: a fast Fourier convolution branch for global feature extraction and a wavelet convolution branch for local feature extraction. Both Fourier and wavelet convolutions provide larger receptive fields compared to conventional convolution methods, thereby enabling the extraction of dual-scale features. Finally, these features are integrated using a dynamic gating fusion module, which enhances the representation of both local and global time–frequency information.

Although both FFC and wavelet convolution can enhance feature representation, they focus on different signal characteristics and therefore provide complementary information. FFC transforms feature maps into the frequency domain and enables interactions over the entire feature map, thereby providing a global receptive field. This property is particularly suitable for capturing long-range dependencies, periodic structures, and order-related fault patterns in rotating machinery signals, especially after angular-domain resampling by HOITR.

In contrast, wavelet transform decomposes the input into low-frequency and high-frequency sub-bands, providing localized multi-scale time–frequency representations. The low-frequency sub-band preserves the main trend and global energy distribution, while the high-frequency sub-bands emphasize transient impulses, local variations, and detailed fault-related components. These localized characteristics are important for identifying incipient faults and weak fault signatures under complex noise interference.

Therefore, the FFC branch and the wavelet branch are complementary rather than redundant. The former focuses on global frequency domain dependencies and periodic structures, whereas the latter focuses on local multi-scale time–frequency details and transient components. The proposed dynamical gated fusion module further exploits this complementarity by adaptively assigning different weights to the two branches according to the input features, thereby improving diagnostic accuracy and robustness under variable operating conditions.

### 3.2. High-Order Interpolative Tracking Resampling (HOITR)

To address this issue, HOITR is introduced before feature extraction. By using rotational speed pulse information, HOITR establishes a mapping from the time domain to the angular domain and resamples the vibration signal according to equal angular intervals. After this transformation, vibration components associated with rotating parts are normalized with respect to the rotation angle. Consequently, the originally nonstationary time-domain signal is converted into a more stable angular-domain representation. This preprocessing step reduces the influence of speed fluctuation and provides a consistent input scale for the subsequent FFC and WTConvNext branches.

**Figure 2:**
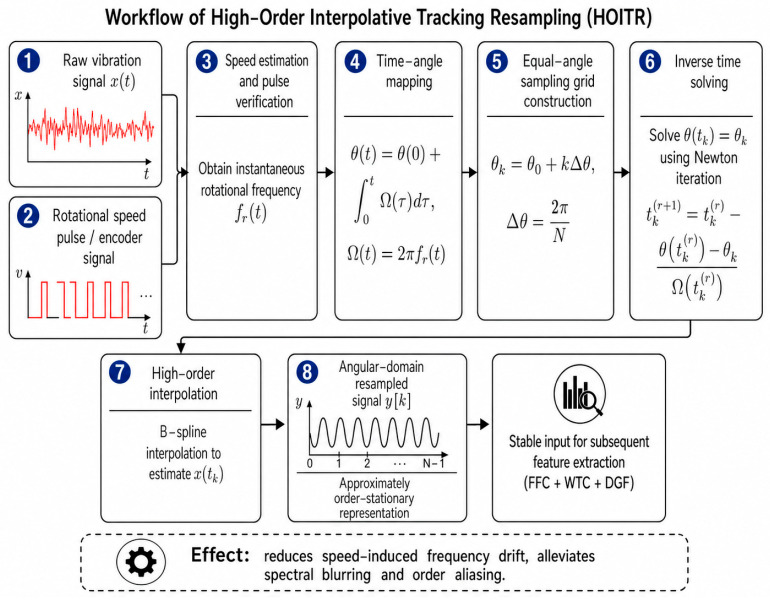
Schematic diagram of high-order interpolative tracking resampling.

As illustrated in Figure 2, the rotational speed pulse is frequency-multiplied and interpolated using a high-order tracking strategy. Through this process, variable-speed nonstationary signals in the time domain are resampled into an approximately order-stationary angular-domain representation.

For rotating machinery operating under variable-speed conditions, the measured vibration signal is generally nonstationary in the time domain. The fault-related characteristic frequencies vary with the instantaneous rotational speed, which may lead to spectral blurring and order aliasing when conventional Fourier analysis is directly applied to the raw time-domain signal. To alleviate this problem, High-Order Interpolative Tracking Resampling (HOITR) is introduced to transform the signal from the time domain into the angular domain. The purpose of HOITR is to normalize the signal with respect to the rotation angle, so that speed-dependent frequency drift can be converted into relatively stable order-domain patterns. To provide a theoretical explanation of HOITR, the measured vibration signal under time-varying speed can be modeled as follows:

(1) $$x(t)=\sum_{m\in M}A_{m}(t)cos(m\theta (t)+\phi_{m})+n(t),$$

where x(t) denotes the measured time-domain vibration signal, *t* is the time variable, M is the set of mechanical orders associated with rotating components, *m* denotes the mechanical order, $A_{m}\left(t\right)$ is the time-varying amplitude of the *m*-th order component, $\phi_{m}$ is the corresponding phase, $n\left(t\right)$ denotes additive noise, and $\theta \left(t\right)$ is the instantaneous angular position of the shaft.

The instantaneous angular position is obtained from the rotational speed as

(2) $$\theta (t)=\theta (0)+\int_{0}^{t}\Omega (\tau )d\tau ,\Omega (t)=2\pi f_{r}(t),f_{m}(t)=mf_{r}(t),$$

where Ω(t) is the instantaneous angular velocity, $f_{r}(t)$ is the instantaneous rotational frequency, and $f_{m}(t)$ is the instantaneous frequency of the *m*-th order component. Equation (2) shows that when $f_{r}(t)$ varies with time, the fault-related frequency $f_{m}(t)$ also drifts in the time domain. Therefore, a component corresponding to a fixed mechanical order may be distributed over a broadened frequency band in the time-domain spectrum.

In HOITR, each rotation cycle is divided into *N* equal angular intervals. The angular sampling grid is defined as

(3) $$\theta_{k}=\theta_{0}+k\Delta \theta ,\Delta \theta =\frac{2\pi }{N},k=0,1,\ldots ,K-1,$$

where $\theta_{0}$ is the initial angular position, Δθ is the angular sampling interval, *N* is the number of angular samples per revolution, and *K* is the total number of angular sampling points.

The corresponding time instant $t_{k}$ of the *k*-th angular sampling point is determined by solving the time–angle mapping equation:

(4) $$\theta (t_{k})=\theta_{k},t_{k}=\theta^{-1}(\theta_{k}).$$

When Ω(*t*) > 0, the angular position *θ*(*t*) is monotonically increasing, and the mapping between t and *θ* is locally invertible. In engineering applications, *θ*(*t*) is estimated from rotational speed pulses or encoder signals. Since the desired angular sampling points usually do not coincide with the original time-domain sampling points, numerical interpolation is required to obtain the corresponding time instants. To solve Equation (4), the Newton–Raphson iteration can be used:

(5) $$t_{k}^{\left(r+1\right)}=t_{k}^{\left(r\right)}-\frac{\theta (t_{k}^{\left(r\right)})-\theta_{k}}{\Omega (t_{k}^{\left(r\right)})},$$

where $t_{k}^{\left(r\right)}$ is the estimate of $t_{k}$ at the *r*-th iteration. The iteration is terminated when the residual $|\theta (t_{k}^{\left(r\right)})-\theta_{k}|$ is smaller than a predefined tolerance. The final estimate is denoted as ${\hat{t}}_{k}$. After obtaining ${\hat{t}}_{k}$, the angular-domain signal is reconstructed by high-order interpolation. In this study, the interpolation process can be written as

(6) $$y[k]=S_{p}\{x(t)\}{|}_{t={\hat{t}}_{k}}=\sum_{i\in N_{k}}c_{i}B_{p}({\hat{t}}_{k}-t_{i}),$$

where y[k] denotes the angular-domain resampled signal, $S_{p}\{\cdot \}$ is the *p*-th order interpolation operator, $B_{p}(\cdot )$ is the B-spline basis function, $c_{i}$ is the interpolation coefficient, $t_{i}$ is the original time-domain sampling point, and $N_{k}$ is the local support set used for interpolation. This formulation is consistent with the high-order interpolation strategy used in the original HOITR procedure. Substituting the equal-angle sampling grid into the *m*-th order component gives

(7) $$y_{m}[k]=A_{m}({\hat{t}}_{k})cos(m\theta_{k}+\phi_{m}).$$

Equation (7) indicates that a component with time-varying frequency in the original time domain is converted into a fixed-order component in the angular domain. Therefore, HOITR does not make the signal strictly stationary in the statistical sense. Instead, it converts the variable-speed time-domain signal into an approximately order-stationary angular-domain representation. In this representation, speed-induced frequency drift is reduced, while the order-related fault patterns are better aligned.

The interpolation and resampling error can be expressed as

(8) $$\varepsilon_{y}[k]=|x(t_{k})-x({\hat{t}}_{k})|\leq L_{x}|t_{k}-{\hat{t}}_{k}|+\varepsilon_{s},$$

where $\varepsilon_{y}[k]$ is the resampling error, $L_{x}$ is the local Lipschitz constant of x(t), and $\varepsilon_{s}$ denotes the error caused by sensor noise, speed pulse measurement error, and acquisition uncertainty. Equation (8) shows that the accuracy of the time–angle mapping directly affects the quality of the reconstructed angular-domain signal. Therefore, pulse verification and high-order interpolation are used to improve the stability of HOITR.

For reliable angular-domain representation, two engineering constraints should be considered:

(9) $$\underset{t\in T}{max}|\frac{\dot{\Omega }(t)}{\Omega (t)}|T_{w}<\gamma ,N\geq 2O_{max},$$

where $T_{w}$ is the local interpolation window, *γ* is a predefined threshold for allowable local speed fluctuation, and $O_{max}$ is the maximum mechanical order to be analyzed. The first condition ensures that the rotational speed does not vary too abruptly within the local interpolation window, thereby improving interpolation stability. The second condition is the angular-domain sampling requirement, which prevents order aliasing according to the Nyquist criterion.

Through the above procedure, HOITR maps the nonstationary vibration signal from the time domain to the angular domain. The resulting representation is not strictly stationary, but it is approximately order-stationary because the main fault-related components are aligned with mechanical orders. This provides a more stable and discriminative input for the subsequent FFC branch, wavelet convolution branch, and dynamical gated fusion module.

### 3.3. Wavelet Convolutional Neural Networks

After HOITR, the input signal is represented in the angular domain, where the main order-related fault components become more stable. However, rotating machinery faults are not only reflected by global periodic order structures, but also by local transient impulses, high-frequency details, and weak time-varying components. Therefore, local multi-scale time–frequency feature extraction is required in addition to global feature modeling.

**Figure 3:**
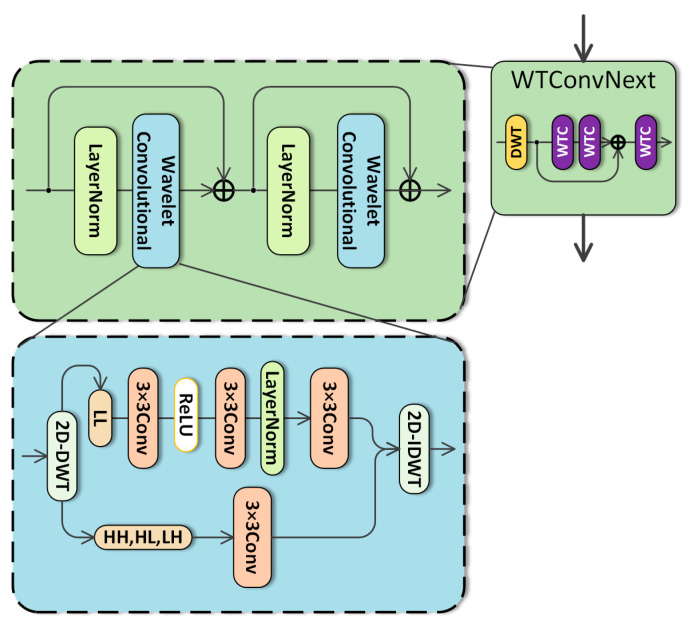
Structure of the Wavelet convolution.

DWT is used to split the input into four sub-bands. The low-frequency sub-band preserves coarse-scale trend and energy distribution, whereas the high-frequency sub-bands capture transient impulses, abrupt variations, and weak fault-related details. For $I\in R^{H\times W\times C}$, we use Haar wavelets for their simplicity and speed. The Haar wavelet transform filters are divided into two types: low-pass L and high-pass H. As illustrated in Figure 3, it can be represented as

(10) $$L=\frac{1}{\sqrt{2}}{\left[1,1\right]}^{T},H=\frac{1}{\sqrt{2}}{\left[1,-1\right]}^{T}$$

The input can then be divided into four sub-bands, which can be stated as follows:

(11) $$I_{LL},\{I_{LH},I_{HL},I_{HH}\}=2D-DWT(I).$$

$I_{LL},I_{LH},I_{HL},I_{HH}\in R^{\frac{H}{2}\times \frac{W}{2}\times C}$. The LL sub-band preserves coarse-scale trend information and the global energy distribution of the feature map, whereas the LH, HL, and HH sub-bands emphasize high-frequency details, including transient impulses, abrupt variations, and weak fault-related components. Compared with conventional downsampling, DWT explicitly separates low-frequency approximation information from high-frequency detail information, which helps preserve weak fault signatures during feature extraction., leading to the loss of some features; conversely, DWT maintains channel count and preserves information due to its bi-orthogonality trait. Subsequently, the IWT procedure is employed to reconstruct the output, which can be articulated as

(12) $$I_{output}=2D-IWT\left(I_{LL},I_{LH},I_{HL},I_{HH}\right).$$

In the proposed framework, time–frequency localization is mainly provided by the wavelet convolution branch. Compared with the Fourier transform, which describes the global frequency distribution of a signal, the wavelet transform provides localized representations in both the position and frequency-scale domains. This property is particularly important for rotating machinery fault diagnosis, because fault-related vibration components often appear as local transient impulses, high-frequency details, and weak time-varying patterns.

In this study, the Haar-based discrete wavelet transform decomposes the input feature map into four sub-bands, namely LL, LH, HL, and HH. The LL component preserves the low-frequency trend and global energy distribution, whereas the LH, HL, and HH components highlight high-frequency details in different directions. These high-frequency sub-bands are beneficial for capturing local impacts, abrupt changes, and weak fault-related components. Therefore, the WTConvNext branch enhances the time–frequency localization capability of the network and provides local multi-scale information that complements the global frequency domain representation extracted by the FFC branch [14].

The feature extraction process of DG-FuseNet is composed of two complementary branches. Let the input feature map at a given layer be denoted as X ∈ R^{B×C×H×W}, where B, C, H, and W represent the batch size, the number of channels, and the two spatial feature dimensions, respectively. In the WTConvNext branch, the discrete wavelet transform is applied to decompose X into four sub-bands, namely LL, LH, HL, and HH, each of which characterizes signal information at a specific frequency scale. Specifically, the LL sub-band corresponds to coarse-scale low-frequency information and mainly preserves the global structural characteristics of the feature map, whereas the LH, HL, and HH sub-bands represent fine-scale high-frequency details in different directions. Through this decomposition, the WTConvNext branch can explicitly separate low-frequency structural information from high-frequency detail information, thereby enhancing the multi-scale feature representation capability of DG-FuseNet.

From the dimensional perspective, the wavelet transform converts the original feature map into multiple sub-band representations with reduced spatial resolution but enhanced frequency-scale diversity. These sub-bands provide multi-scale features for subsequent convolutional extraction. After wavelet-domain processing, inverse wavelet transform is used to reconstruct the feature representation. In parallel, the FFC branch extracts local and global features by combining standard local convolution with global frequency domain convolution. Finally, the dynamical gated fusion module adaptively integrates the FFC and WTConvNext features, allowing the model to jointly use global periodic dependencies and local multi-scale fault details.

### 3.4. Fast Fourier Convolution Neural Networks

**Figure 4:**
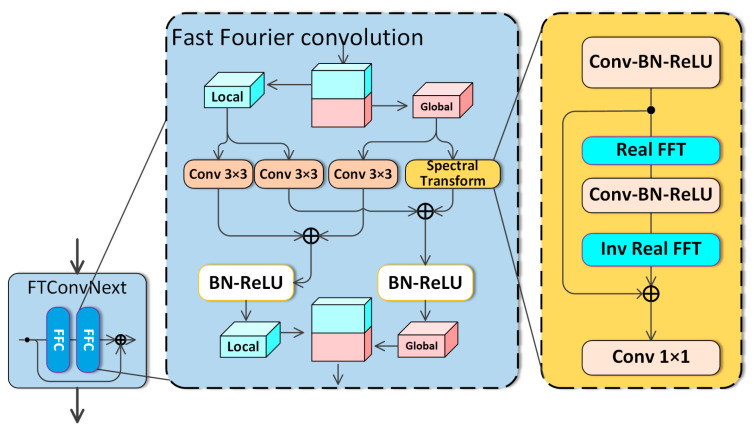
Structure of the Fast Fourier convolution.

Fast Fourier convolution converts standard spatial domain convolution to the frequency domain. It uses the equivalence of discrete convolution in the frequency domain (i.e., element-by-element multiplication) to create a global receptive field that matches high-order sampling samples. Recent operators like fast Fourier convolution (FFC) [10] offer global context in early layers. As illustrated in Figure 4, FFC uses Fourier transform properties to combine local and global convolution in one operation unit. The Fourier transform converts feature maps from the spatial/angular domain into the frequency domain.

A global receptive field is theoretically possible since a single pixel update affects all pixels in the frequency domain. FFC uses a channel-wise fast Fourier transform (FFT) and features a global receptive field. FFC divides channels into two parallel branches: local utilizes standard convolutions, while global employs real FFT for global context. Real FFT only works on real signals, and inverse real FFT guarantees real output. Compared to FFT, real FFT consumes half the spectrum. Specific steps taken by FFC include.

Applying Real FFT2d on the input tensor

(13) $$RealFFT2d:R^{H\times W\times C}\to C^{H\times \frac{W}{2}\times C}$$

The real and imaginary parts are concatenated:

(14) $$ComplexToReal:C^{H\times \frac{W}{2}\times C}\to R^{H\times \frac{W}{2}\times 2C}$$

Use a frequency domain convolution block:

(15) $$ReLU\circ BN\circ Conv1\times 1:R^{H\times \frac{W}{2}\times 2C}\to R^{H\times \frac{W}{2}\times 2C}$$

Recover spatial structure using inverse transform:

(16) $$RealToComplex:R^{H\times \frac{W}{2}\times 2C}\to C^{H\times \frac{W}{2}\times C}$$

(17) $$InverseRealFFT2d:C^{H\times \frac{W}{2}\times C}\to R^{H\times W\times C}$$

Finally, the local (i) and global (ii) branch outputs are combined. FFC is shown in Figure 4. Power of FFCs: They are simple replacements for traditional convolutions and are fully differentiable. The global receptive field of FFC enables the model to capture long-range dependencies and order-related periodic structures in angular-domain vibration representations. This is particularly useful for rotating machinery fault diagnosis, because fault-related components often appear periodically over multiple rotation cycles. This improves feature interaction efficiency because global information can be modeled directly in the frequency domain rather than being propagated gradually through many stacked local convolution layers, and rather than waiting for information to propagate. FFCs are particularly effective at capturing periodic structures, which are common in rotating equipment problem detection.

### 3.5. Dynamic Gated Feature Fusion

**Figure 5:**
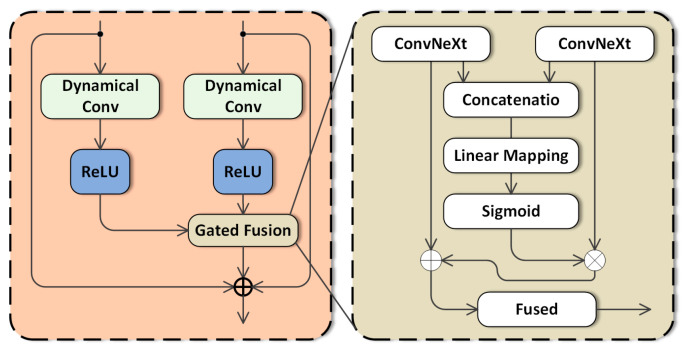
Structure of the Dynamic Gated Feature Fusion module.

As illustrated in Figure 5, the proposed DGF module adaptively integrates the FFC and WTConvNext features. The proposed Dynamic Gated Feature Fusion (DGF) module adaptively adjusts the contributions of the FFC and WTConvNext branches according to the input features. Unlike fixed fusion strategies such as direct addition or concatenation, DGF generates sample-specific gating coefficients to balance global order-related features and local multi-scale time–frequency features, overcoming the drawbacks of previous techniques. Integrating the multi-layer features of fast Fourier convolutional branches and wavelet convolutional branches effectively is essential for enhancing model performance. To address this, Dynamical Gated Fusion (DGF) is introduced as a new feature fusion approach inspired by the attention mechanism and gated mechanism. DGF seeks to improve the model’s performance by adaptively adjusting the weighting coefficients of input features, particularly in complex tasks and multimodal data processing.

The FFC branch and the wavelet branch extract different but complementary features. The FFC branch focuses on global order-related periodic structures and long-range dependencies, whereas the wavelet branch focuses on local multi-scale time–frequency details and transient fault components. However, the importance of these two types of features may vary across different fault modes, operating conditions, noise levels, and signal quality. Therefore, a fixed fusion strategy, such as direct addition or concatenation, may not be optimal for all input samples.

To address this problem, a Dynamic Gated Feature Fusion (DGF) module is introduced to adaptively integrate the features extracted by the two branches. Let

(18) $$F_{FFC}\in R^{B\times C\times H\times W}$$

and

(19) $$F_{WT}\in R^{B\times C\times H\times W}$$

denote the feature representations extracted by the FFC branch and the wavelet branch, respectively. Before fusion, the two feature maps are aligned to the same dimensional space using convolutional projection when necessary.

The two feature maps are first concatenated along the channel dimension:

(20) $$F_{cat}=Concat\left(F_{FFC},F_{WT}\right),$$

where

(21) $$F_{cat}\in R^{B\times 2C\times H\times W}.$$

Global average pooling is then applied to obtain a compact channel-wise descriptor:

(22) $$z=GAP(F_{cat}),$$

where $GAP\left(\cdot \right)$ denotes global average pooling, and

(23) $$z\in R^{B\times 2C}.$$

The adaptive gating coefficient is generated using a lightweight multi-layer perceptron:

(24) $$G=\sigma (W_{2}\delta (W_{1}z+b_{1})+b_{2}),$$

where $W_{1}$, $W_{2}$, $b_{1}$, and $b_{2}$ are learnable parameters, δ(·) denotes the nonlinear activation function, σ(·) is the sigmoid activation function, and *G* is the adaptive gating coefficient. The value of *G* is constrained to the range [0, 1], and it is broadcast to match the spatial dimensions of the feature maps during fusion.

The dynamically fused feature representation is computed as

(25) $$F_{fused}=G⊙F_{FFC}+(1-G)⊙F_{WT},$$

where ⊙ denotes element-wise multiplication. Equation (25) shows that the DGF module performs input-adaptive feature fusion. When global order-related periodic information is more discriminative, the gate assigns a larger weight to the FFC branch. Conversely, when local transient impulses or high-frequency fault details are more important, the gate assigns a larger weight to the wavelet branch.

Compared with static fusion strategies, DGF has two main advantages. First, it adaptively adjusts the contribution of each branch according to the input sample, which improves robustness under variable operating conditions. Second, it reduces redundant or noisy feature responses by emphasizing the more informative branch for each sample. This is particularly important for real industrial vibration signals, where speed fluctuation, load variation, control-system disturbance, and environmental noise may cause significant differences in feature distributions.

After dynamic fusion, the fused feature map is fed into the classifier. The class probability distribution is obtained as

(26) $$p=Softmax(W_{c}GAP(F_{fused})+b_{c}),$$

where $W_{c}$ and $b_{c}$ are the parameters of the classifier, and *p* denotes the predicted probability distribution over all fault classes.

The model is trained using the cross-entropy loss function:

(27) $$L=-\frac{1}{B}\sum_{i=1}^{B}\;\sum_{c=1}^{C_{f}}y_{i,c}log(p_{i,c}),$$

where *B* is the batch size, $C_{f}$ is the number of fault classes, $y_{i,c}$ is the one-hot label of the *i*-th sample for class *c*, and $p_{i,c}$ is the predicted probability of the *i*-th sample belonging to class *c*.

Overall, the DGF module provides a theoretical and practical mechanism for integrating global and local multi-scale features. By adaptively balancing the FFC and wavelet branches, DG-FuseNet can better handle multiple failure modes, variable operating conditions, and noise interference in rotating machinery fault diagnosis.

## 4. Experimental Verification and Comparison Analysis

Although some studies have addressed certain key issues to some extent, most models, when evaluated using datasets, are often limited to simple experimental conditions and have not been thoroughly tested on data from actual industrial production equipment. This study selected two complex mechanical systems to verify the algorithm’s feasibility and potential for industrial application. The first dataset comes from the automatic fault diagnosis system of the running gear of Shanghai Metro vehicles in China. This system has been collecting fault data of rotating mechanical components such as bearings, gears, and wheels of the running gear since 2014. These data record the entire life cycle information of the mechanical parts of the running gear and rotating parts of metro vehicles since their manufacture. The second dataset is from the aero-engine simulation test bench. The data reveals that rotors and blades across every engine level contain pronounced characteristic information, which significantly complicates achieving accurate fault diagnoses. Compared with ordinary datasets, these two datasets have more complex rotor components and environmental noise, providing a highly valuable verification basis for the development of algorithms with industrial application prospects.

### 4.1. Dataset Description

#### 4.1.1. Dataset A: Metro Train Running Gear Rotating Machinery Dataset (MTRG)

**Figure 6:**
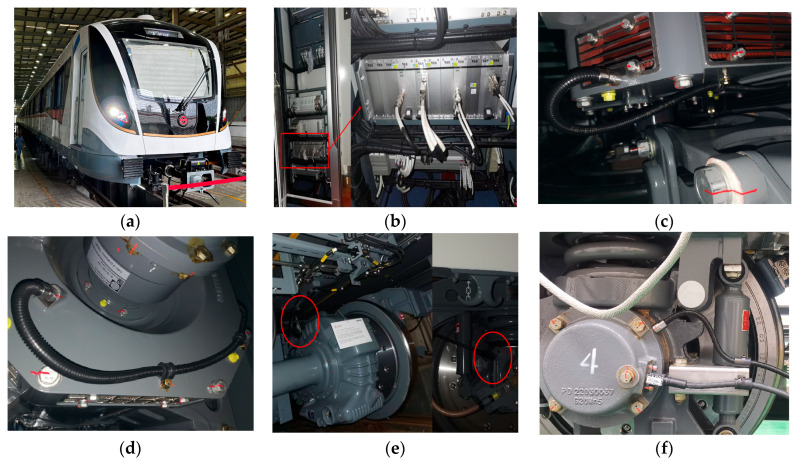
Metro vehicle running gear monitoring system. (**a**) Shanghai metro rolling stock; (**b**) Tangzhi Technology JK16450 (Changsha, China) online fault diagnosis device; (**c**) Motor non-drive end measuring point; (**d**) Motor drive end measuring point; (**e**) Pinion gear motor-side and wheel-side measuring points; (**f**) Wheelset, axle box measuring point.

This dataset is derived from the online monitoring system installed on the running gear (i.e., the bogie system) of metro vehicles shown in Figure 6. This system is deployed on metro vehicles and is responsible for obtaining real-time vibration, shock and temperature data of axle box bearings, motor drive bearings, non-drive end bearings of motors and gearboxes during operation. The sampling rate was 40 kHz, and the rotational speed pulses and the shock and vibration data of the measurement points were collected synchronously.

The traction motor is manufactured by Siemens AG (Munich, Germany), model ZXAM1901A, with a power rating of 190 kW, a rated speed of 1800 rpm, and a maximum speed of 4350 rpm. The motor is connected to the gearbox by a crowned gear coupling. The speed sensor is manufactured by Knorr-Bremse AG (Munich, Germany), model FS01A (see Table 1).

**Figure 7:**
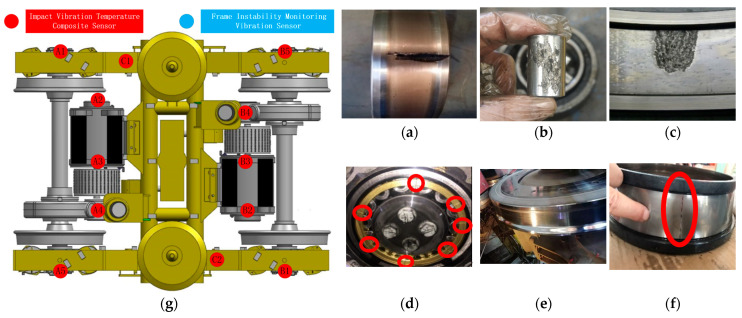
Failure cases of the MTRG dataset; (**a**) inner ring fault1; (**b**) roller failure; (**c**) outer ring failure; (**d**) QJ bearing failure; (**e**) Tread Polygon failure; (**f**) inner ring failure2; (**g**) subway vehicle bogie running system.

The layout of the sensor monitoring points on the bogie is detailed in Figure 7. In this study, we selected data from six measurement points and their corresponding six failure modes. The selected records include real fault cases and normal operating data collected during regular service. After the cleaning process described below, abnormal and invalid records were removed. The MTRG dataset was cleaned through a multi-step quality-control procedure. First, data integrity was checked, and records with missing values, duplicated timestamps, incomplete sequences, or abnormal file lengths were removed. Second, sensor validity was examined by excluding records with sensor saturation, abnormal zero drift, long-term constant output, or physically unreasonable amplitude ranges. Third, the rotational speed pulse sequence was verified, and records with pulse loss, duplicated pulses, or discontinuous speed trajectories were removed. Fourth, operating-condition consistency was checked to exclude non-target operating periods, maintenance periods, shutdown intervals, and abnormal impact events unrelated to the considered fault modes. Fifth, signal quality was screened using statistical indicators such as RMS, peak-to-peak value, kurtosis, and median absolute deviation. Finally, the fault labels were cross-checked using maintenance records, inspection reports, and expert confirmation to reduce the risk of mislabeled samples.

**Figure 8:**
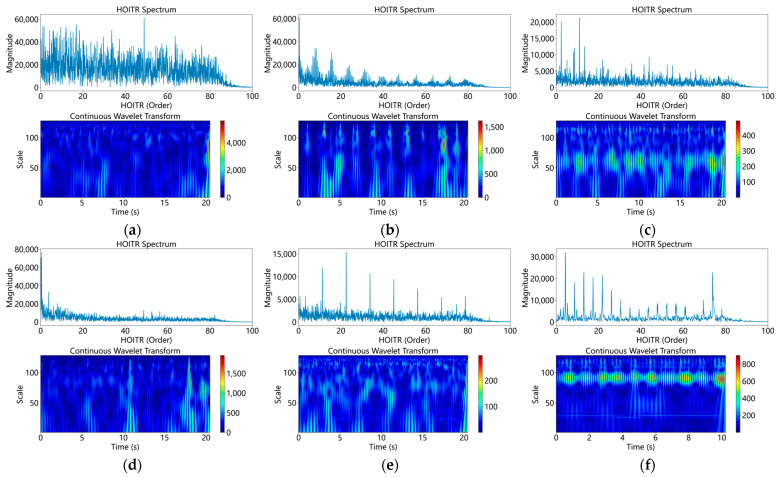
Original data display of the MTRG dataset. (**a**) Inner ring fault1; (**b**) roller failure; (**c**) outer ring failure; (**d**) QJ bearing failure; (**e**) tread polygon failure; (**f**) inner ring failure2.

The feature maps associated with the fault cases in our dataset indicate that subway vehicle failures exhibit diverse and complex characteristics, which cannot be effectively modeled using traditional fault mode theories alone. The fault cases selected for this study are derived from real-world instances, reflecting the varied nature of bearing faults observed in industrial settings. For example, a fault in a bearing is not confined to a single component, such as the inner ring, rollers, or outer ring; some bearings exhibit significant inner ring failures, whereas others display prominent roller defects. Additionally, the train vehicles’ running gear incorporates specialized QJ bearings and multipolygonal wheel treads. The long-term accumulation of field data has resulted in a dataset that encompasses these diverse fault categories, forming one of the core datasets analyzed in this project (see Figure 8).

#### 4.1.2. Dataset B: Harbin Institute of Technology Aero-Engine Bearing Dataset (HIT)

**Figure 9:**
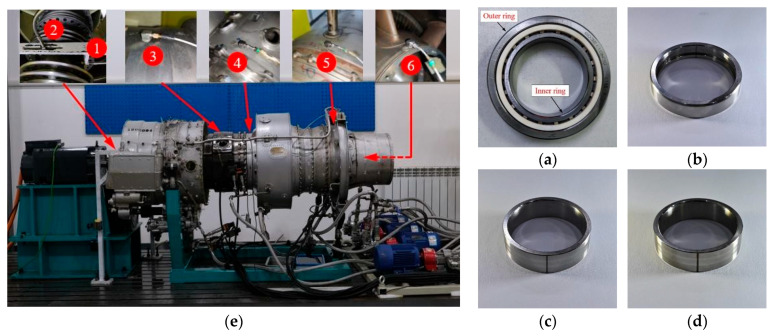
HIT test rig and fault bearing samples. (**a**) Normal bearing; (**b**) outer ring failure; (**c**) inner ring level one fault; (**d**) inner ring level two fault; (**e**) The test rig based on a real aero-engine.

**Figure 10:**
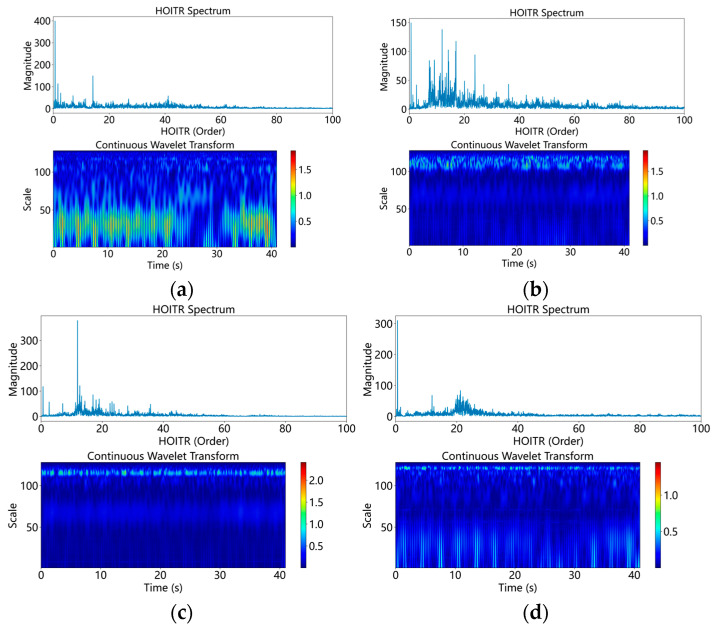
Representative raw signals of the HIT dataset. (**a**) Normal bearing; (**b**) outer ring failure; (**c**) inner ring level one fault; (**d**) inner ring level two fault.

The test rig consists of a modified aero-engine, a motor drive system, and a lubrication system [15]. The core component, a modified aero-engine, produces vibration signals. Additionally, the motor drive system varies the engine’s speed and load. Meanwhile, the lubrication system is vital for maintaining engine efficiency. Figure 9 illustrates the physical layout of the test rig. Figure 10 demonstrates that data from industrial operating sites often contains extensive and complex time–frequency information that standard defect diagnosis models cannot interpret.

The artificial faults were created by wire cutting, as shown in Figure 9a–d, and the faulty bearing data, normal bearing data, and the label list are detailed in Table 2. The displacement and acceleration sensors collect signals and input them through the TRION-2402-dACC differential multifunctional module. These signals are analyzed by the DEWETRON DEWE2-M7 (Graz, Austria) and saved on the computer for later analysis. The dual-rotor aero-engine test rig is shown in Figure 9e. Two eddy current sensors are used to measure the horizontal and vertical vibration displacement response of the LP rotor. Four acceleration sensors are used to measure the normal vibration acceleration response of the casings; the information of the sensors is shown in Table 3. The number of testing points corresponds to the channel number in the dataset [15].

The redesigned test rig’s aero-engine removes the rotor blades, combustion chamber, and auxiliary casings. The core element, the dual-rotor configuration, remains unchanged.

This configuration includes LP and HP compressors and LP and HP turbines. The main load-bearing casing, inter-shaft bearing, and five support bearings are still present. Representative raw vibration responses under different bearing states are shown in Figure 10. Raw data is collected every 15 s, with a sampling rate of 25 kHz. Each 15 s raw record was segmented into fixed-length samples with a window length of 1024 points and an overlap ratio of 50%, consistent with the settings in Table 4. The selected displacement and acceleration channels were normalized using z-score normalization before being input into the model.

### 4.2. Experimental Design and Setup

#### 4.2.1. Experimental Design

The experiment’s software environment includes Ubuntu 22.04, Miniconda 3, Py-thon 3.10, and CUDA 11.8. The hardware platform was equipped with an NVIDIA RTX 4090 GPU (Santa Clara, CA, USA) with 24 GB of memory and an Intel Xeon Platinum 8352V CPU (12 vCPUs, 2.10 GHz) (Santa Clara, CA, USA).

After establishing DG-FuseNet according to the specifications in Figure 1 and dividing the dataset into training and testing subsets, the next step is to configure the model’s training parameters. The setup involves selecting a learning rate of 0.001, using the Adam optimizer for parameter updates, and choosing the classification cross-entropy as the loss function. Additionally, the batch size is set to 64 for each training iteration. After completing each training cycle, the model’s diagnostic performance is evaluated using the test dataset to verify its accuracy. To ensure reproducibility and reduce the influence of random initialization, all experiments were repeated five times with different random seeds, i.e., 2021, 2022, 2023, 2024, and 2025. These seeds were used to control data partitioning, model initialization, mini-batch shuffling, and noise generation. The final results are reported as the mean value and standard deviation over the five independent runs. The remaining detailed parameters are listed in Table 4.

#### 4.2.2. Compare the Schemes with Other Advanced Models

To assess the effectiveness of the proposed technique in the fault detection of aero-engine intermediate bearings, we selected eleven state-of-the-art methods. We performed comparative experiments using rotating components from train vehicle running gear alongside publicly available datasets of aero-engine intermediate bearings.

Among these approaches, 1D_2DIFCNN [16] and CFCNN [17] are used as baseline methods. In contrast, advanced algorithms for time series classification, ACEL [18], DNOCNet [19], PGCNN [20], DRCNN [21], ATCATN [22], and DGE-GNN [23], are employed in the comparative experiments. In this study, we compare DG-FuseNet with eleven competing approaches.

To evaluate the system’s resilience to noise, we add Gaussian white noise to create a noisy signal. The signal-to-noise ratio is defined as follows:

(28) $$SNR=10log(\frac{P_{signal}}{P_{noise}})$$

Among them, *Psignal* represents the power of the original signal, and *Pnoise* represents the power of the noise.

To ensure a comprehensive and persuasive comparison, the selected methods cover several representative categories of intelligent fault diagnosis models. Specifically, CNN-based and residual models were selected as classical deep learning baselines for vibration signal diagnosis. Attention-based and lightweight models were included to evaluate the effectiveness of feature enhancement and efficient representation learning. Graph-based and variable-condition models were selected because they are suitable for complex operating conditions and cross-domain diagnosis. In addition, CFCNN and 1D_2DIFCNN were included as feature-fusion baselines, since they are closely related to the fusion strategy considered in this study.

It should be noted that although some compared methods employ multi-scale feature extraction, attention mechanisms, denoising strategies, or feature fusion, the implemented comparison models do not explicitly integrate wavelet sub-band decomposition with FFC-based global convolution and dynamical gated fusion. In contrast, the proposed DG-FuseNet jointly exploits global frequency domain dependencies from the FFC branch, local multi-scale time–frequency features from the wavelet branch, and adaptive feature weighting from the dynamical gated fusion module. Therefore, the selected comparison methods provide a representative and fair basis for evaluating the effectiveness of the proposed framework. Table 5 summarizes the core mechanisms of the comparative methods.

### 4.3. Experimental Results and Analysis

#### 4.3.1. Experimental Results of Two Datasets

**Figure 11:**
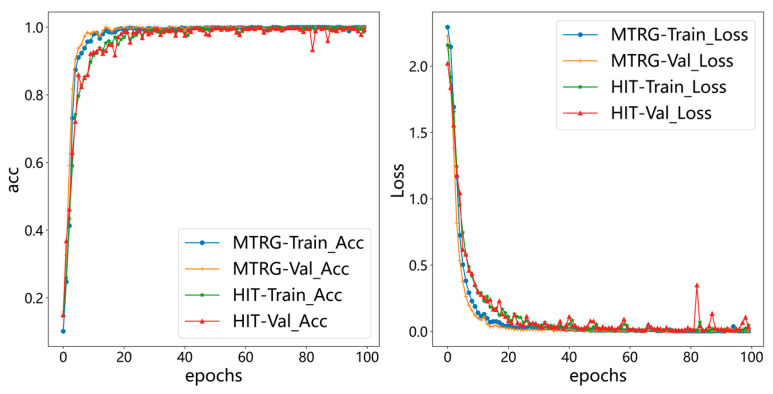
Training and validation loss graphs of two dataset models.

As shown in Figure 11, training was conducted separately on the MTRG and HIT datasets, and the model’s performance was evaluated using accuracy and loss curves. As illustrated by the accuracy curves (left graph), both the training and validation accuracies for the MTRG and HIT datasets gradually stabilized over successive epochs, converging toward similar values. Similarly, the loss curves (right graph) show that the training and validation losses for both datasets consistently decreased to low levels and then plateaued, indicating that the model successfully converged on both datasets.

**Figure 12:**
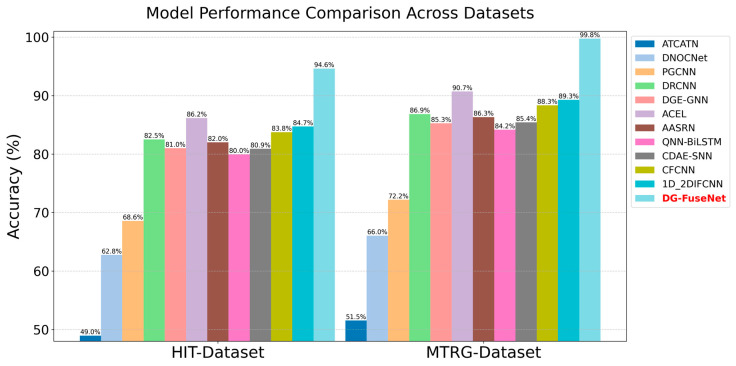
Diagnostic accuracy of the proposed method and eleven comparison methods.

To compare the performance of various models in fault classification tasks on real-world industrial datasets, this study selected two types of datasets—HIT and MTRG—and tested DG-FuseNet and eleven comparison methods. As shown in Figure 12, the horizontal axis represents the data source (HIT-Dataset, MTRG-Dataset), while the vertical axis indicates the accuracy, ranging from 0 to 100%, which measures the proportion of correctly classified cases; the higher the value, the better the performance. The legend displays the performance comparison of the DG-FuseNet and eleven comparison methods, such as ATCATN, DNOCNet, and DG-FuseNet, with each model represented by a bar of a different color.

**Figure 13:**
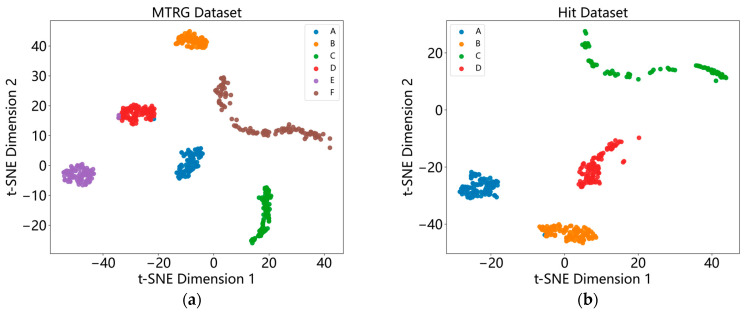
Model TSNE visualization. (**a**) MTRG dataset; (**b**) HIT dataset.

As shown in Figure 13, t-Distributed Stochastic Neighbor Embedding (t-SNE) is a dimensionality reduction visualization method used to map high-dimensional data to two- or three-dimensional space to observe the data distribution. The following analysis examines the model’s classification performance on the two datasets based on their t-SNE distributions.

**Figure 14:**
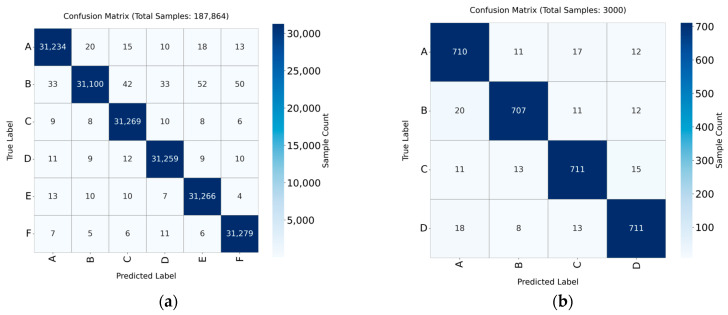
Confusion Matrix Diagram of Model Classification Performance. (**a**) Confusion Matrix Diagram of MTRG dataset; (**b**) Confusion Matrix Diagram of HIT dataset.

As shown in Figure 14, the effectiveness of the model on real-world data, where the dataset reflects actual operating scenarios, is demonstrated through quantitative analysis using confusion matrices. Two confusion matrices (MTRG, HIT–Dataset) display the distribution of correct and incorrect classification results, thereby highlighting the model’s performance. The focus is on the proportion of diagonal entries (indicating correct classifications) and the misclassification cases. Comparative experiments on the training process of different methods were performed.

**Figure 15:**
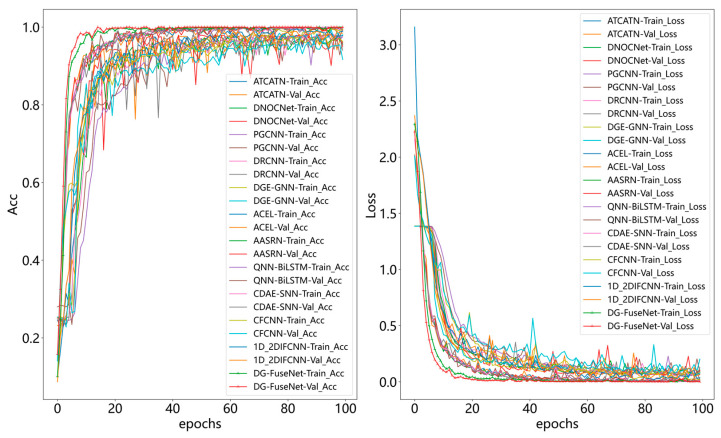
Comparative experiments on the performance of different methods.

As shown in Figure 15, a comparison of the training loss curves recorded during the training of DG-FuseNet and eleven comparison methods (right figure) indicates that DG-FuseNet exhibits a steeper decline in its training loss, achieving a rapid reduction in loss within fewer epochs and entering a stable loss phase earlier than the other models. Moreover, its loss curve shows minimal fluctuations, consistently remaining within a low-loss region, which demonstrates faster convergence and superior stability. This evidence confirms that the model can efficiently learn data features and accurately optimize errors, significantly outperforming the comparative models in terms of training efficiency and robustness.

#### 4.3.2. Comparative Experiments on the Robustness Against Noisy Scenarios of Different Methods

**Figure 16:**
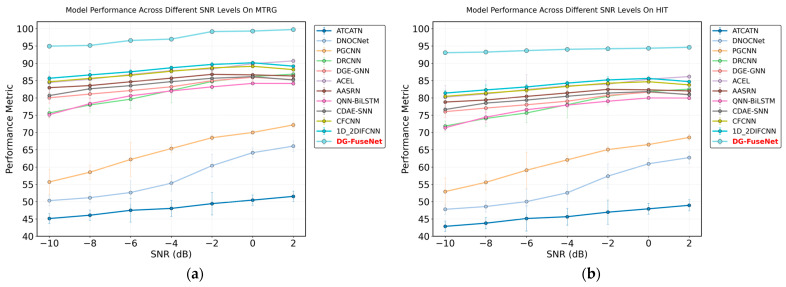
Diagnostic accuracy of the proposed method and eleven comparative methods under noisy scenarios. (**a**) MTRG dataset; (**b**) HIT dataset.

We simulated seven groups of noise scenarios to evaluate the anti-noise robustness of the proposed DG-FuseNet. Specifically, a noise dataset was constructed with signal-to-noise ratios (SNRs) ranging from –10 dB to 2 dB. Notably, when the SNR is –10 dB, the noise energy is ten times greater than the original signal energy, clearly indicating that noise has a substantial impact on diagnostic performance. As expected, higher noise levels correspond to lower diagnostic accuracy across the compared models. Nevertheless, the proposed DG-FuseNet consistently outperforms the other eleven models, demonstrating superior robustness under severe noise conditions. As shown in Figure 16, the robustness evaluation of DG-FuseNet on the MTRG and HIT datasets demonstrates that DG-FuseNet (represented by the red line) consistently outperforms all comparative models across all SNR levels (–10 to 2 dB).

Moreover, as the SNR increases (indicating reduced noise), its performance improves steadily, underscoring its strong robustness and ensuring consistently high accuracy regardless of noise intensity. At an SNR of –10 dB (the maximum noise level), other models, such as ATCATN and PGCNN, suffer a significant performance decline (approximately 45 and 55, respectively), whereas DG-FuseNet maintains exceptionally high performance. These results highlight its outstanding robustness against noise interference, as its feature extraction and classification processes remain largely unaffected by noise.

Furthermore, the standard deviation of DG-FuseNet ranges from 0.10 to 0.29 and from 0.13 to 0.31, with mean values between 0.21 and 0.24, which are significantly smaller than those of the other eleven models. This indicates that, across different noise environments, the accuracy of DG-FuseNet fluctuates less and its performance remains more stable. Therefore, it can be concluded that DG-FuseNet surpasses the other eleven methods in terms of both stability and noise robustness. Detailed test data are listed in Table 6 and Table 7.

#### 4.3.3. Ablation Study on the Integration of FFC and WTConvNext

To further verify the effectiveness of integrating FFC and wavelet convolution, an ablation study was conducted on both the MTRG and HIT datasets. All variants were trained under the same experimental settings, including the same data partitioning strategy, optimizer, learning rate, batch size, and training epochs. The evaluated variants included the following: (1) a baseline convolutional model without FFC or wavelet convolution; (2) an FFC-only model; (3) a WTConvNext-only model; (4) an FFC + WTConvNext model using element-wise addition; (5) an FFC + WTConvNext model using feature concatenation; and (6) the complete DG-FuseNet with dynamical gated fusion. The detailed ablation study models are presented in Table 8.

As shown in Table 9, both the FFC-only and WTConvNext-only variants outperform the baseline-CNN model, indicating that global frequency domain modeling and local multi-scale wavelet representation are individually effective for rotating machinery fault diagnosis. Specifically, The FFC-only model improves the diagnostic accuracy by 28.59 and 33.11 percentage points on the MTRG and HIT datasets, respectively. The WTConvNext-only model improves the diagnostic accuracy by 40.10 and 41.80 percentage points on the MTRG and HIT datasets, respectively. These results suggest that the FFC branch can effectively capture long-range periodic dependencies, whereas the wavelet branch is beneficial for extracting localized multi-scale time–frequency features.

Furthermore, the models combining FFC and WTConvNext achieve better performance than either single-branch model, demonstrating the complementarity between global frequency domain features and local multi-scale wavelet features. Compared with static addition and concatenation fusion, the proposed DG-FuseNet achieves the highest diagnostic accuracy, reaching 99.76% on the MTRG dataset and 94.32% on the HIT dataset. In addition, DG-FuseNet also obtains the best average accuracy under noisy conditions, further confirming its robustness against noise interference.

These results demonstrate that the integration of FFC and wavelet convolution is not a redundant combination. Instead, the two branches provide complementary feature representations, and the dynamic gated fusion module can adaptively adjust their contributions according to the input samples, thereby improving both diagnostic accuracy and robustness under complex industrial operating conditions.

## 5. Conclusions

This paper proposes an innovative dual-scale dynamic gating fusion framework, which performs well under variable-speed conditions. This framework incorporates high-order interpolation tracking resampling (HOITR) technology, which addresses the difficulty of scale normalization under variable rotational speeds. By resampling variable-speed nonstationary signals from the time domain into the angular domain, HOITR converts the raw vibration signals into an approximately order-stationary representation. This transformation reduces the frequency drift caused by speed fluctuations and provides more stable and discriminative input for subsequent feature extraction. Secondly, a dual-branch feature extraction method is adopted, combining the fast Fourier convolution (FFC) branch and the wavelet convolution (WTConvNext) branch to comprehensively capture local and global time–frequency information. The fast Fourier convolution branch realizes the conversion from spatial domain convolution operations to frequency domain multiplication operations through Fourier transform, possesses a global receptive field, and effectively captures long-distance dependencies and periodic structures. Wavelet convolutional branching utilizes wavelet transform for multi-scale time–frequency localization analysis and is adept at capturing edge and texture information. The framework also introduces a dynamic gated fusion (DGF) module, which adaptively adjusts the input feature weight coefficients, effectively fuses dual-scale features, and significantly improves model performance, especially when dealing with complex tasks and multimodal data, whereby it performs exceptionally well. This research provides a new and efficient fusion framework for the field of signal processing and offers a powerful tool for addressing various challenges in actual working conditions.

The relevant design and verification performance demonstrate that the practical application of DG-FuseNet in complex and variable noise environments is very promising. It can deliver more reliable and efficient fault diagnosis solutions for industrial diagnostic tasks. Although the proposed model demonstrates strong performance, its potential limitations must be acknowledged. In deep learning-based fault diagnosis, challenges such as overfitting, hyperparameter optimization, and deployment in dynamic industrial environments remain significant. To address these challenges, strategies including data augmentation, comprehensive robustness testing, and transfer learning can be employed. Accordingly, future research should focus on constructing broader bearing fault datasets under diverse operating conditions to better assess the model’s generalizability and wide applicability.

Although the proposed DG-FuseNet achieves strong diagnostic performance, the wavelet basis used in the current WTConvNext branch is predefined. In future work, we will further investigate task-adaptive wavelet construction, such as learnable Morlet wavelet kernels or order-adaptive wavelet bases, so that the wavelet parameters can be optimized according to the characteristics of rotating machinery fault signals. This may further improve the flexibility and diagnostic capability of the proposed framework under more complex nonstationary operating conditions.

## Figures and Tables

**Table 1:** MTRG data description and labels.

Labels	Object	Fault Type	Measure Point	Bear Model	Manufacturer
A	Motor (Drive End) Bearing	Inner ring fault	A3, B3	6216-85	FAG (Schweinfurt, German)
B	Motor (Non-drive End) Bearing	Roller failure	A2, B2	NU210-65	FAG
C	Pinion Gear (Motor-Side) Bearing	Outer ring failure	A4, B4	NJ216	SKF (Gothenburg, Switzerland)
D	Pinion Gear (Wheel-Side) Bearing	QJ bearing failure	A4, B4	NJ216	SKF
E	Wheelset Tread	Tread Polygon	A1, A5, B1, B5	—	CRRC (Changchun, China)
F	Axle Box Bearing	Inner ring failure	A1, A5, B1, B5	F-587885	FAG

**Table 2:** HIT data description and labels.

Labels	Fault Type	Depth of Fault/mm	Length of Fault/mm
A	Normal	—	—
B	Outer ring	0.5	0.5
C	Inner ring	0.5	0.5
D	Inner ring	0.5	1.0

**Table 3:** Information of sensors.

Sensor Type	Sensor Model	Measure Point
Displacement	KISTLER 8776A50M1 (Winterthur, Switzerland)	1, 2
Acceleration	K9000XL	3, 4, 5, 6

**Table 4:** Detailed experimental settings for reproducibility.

Item	Setting	Item	Setting
Framework	PyTorch 2.1.0	Normalization	z-score
Epochs	100	Batch size	64
Loss function	Cross-entropy loss	Optimizer	Adam
Repeated runs	5 independent runs	Learning rate	0.001
Train/validation/test split	70/10/20	Window overlap	50%
Evaluation metrics	Accuracy, confusion matrix, t-SNE, noise robustness	Window length	1024
Noise levels	−10, −8, −6, −4, −2, 0, 2 dB	Python environment	3.10

**Table 5:** Similar mechanisms are included in the compared model description.

Method	Main Mechanism	WTConvNext	FFC	DGF	Reason for Selection
ATCATN [22]	Attention-based/noise-robust diagnosis	No	No	No	Strong noise-robust baseline
DNOCNet [19]	End-to-end diagnosis under speed fluctuation	No	No	No	Variable-speed baseline
PGCNN [20]	CNN-based fault feature extraction	No	No	No	Classical CNN diagnosis baseline
DRCNN [21]	Deep residual CNN	No	No	No	Residual learning baseline
DGE-GNN [23]	Dynamic graph embedding	No	No	No	Graph-based variable-condition baseline
ACEL [18]	Adaptive multi-scale CNN/LSTM-type model	No/not explicit	No	No	Sequential/multi-scale baseline
AASRN [24]	Attention-augmented separable residual network	No	No	No	Lightweight attention baseline
QNN-BiLSTM [25]	Quadratic neural network with BiLSTM	No	No	No	Temporal dependency baseline
CDAE-SNN [26]	Denoising autoencoder + Siamese network	No	No	No	Denoising/small-sample baseline
CFCNN [17]	Convolutional fusion framework	No/not explicit	No	No	Feature-fusion baseline
1D_2DIFCNN [16]	1D–2D dual-track feature fusion CNN	No/not explicit	No	No	Dual-input feature-fusion baseline
DG-FuseNet	FFC + WTConvNext + DGF	Yes	Yes	Yes	Proposed method

**Table 6:** Diagnostic results of MTRG datasets under different signal-to-noise ratios (%).

Symbol	−10 dB	−8 dB	−6 dB	−4 dB	−2 dB	0 dB	2 dB
ATCATN [22]	45.12 ± 1.42	46.08 ± 1.49	47.50 ± 3.41	48.05 ± 2.33	49.43 ± 3.28	50.45 ± 1.47	51.53 ± 1.52
DNOCNet [19]	50.30 ± 1.38	51.15 ± 1.45	52.65 ± 3.37	55.33 ± 2.29	60.40 ± 3.22	64.16 ± 1.43	66.05 ± 1.48
PGCNN [20]	55.70 ± 3.72	58.51 ± 2.08	62.20 ± 4.89	65.35 ± 1.12	68.48 ± 0.72	70.01 ± 0.48	72.18 ± 0.79
DRCNN [21]	75.64 ± 1.39	77.95 ± 2.07	79.62 ± 2.64	82.13 ± 3.57	84.75 ± 2.47	86.17 ± 0.75	86.86 ± 0.40
DGE-GNN [23]	80.03 ± 1.57	81.10 ± 1.26	82.12 ± 1.08	83.20 ± 1.27	84.96 ± 1.49	85.96 ± 1.09	85.27 ± 1.38
ACEL [18]	84.44 ± 3.11	85.40 ± 3.71	86.75 ± 4.02	87.87 ± 0.76	88.39 ± 0.77	89.88 ± 0.89	90.70 ± 0.67
AASRN [24]	82.95 ± 2.37	83.56 ± 0.71	84.66 ± 1.13	85.70 ± 0.75	86.80 ± 0.78	86.65 ± 0.31	86.33 ± 0.60
QNN-BiLSTM [25]	75.14 ± 0.80	78.33 ± 1.41	80.61 ± 0.19	82.01 ± 0.69	83.19 ± 0.49	84.19 ± 0.36	84.16 ± 0.50
CDAE-SNN [26]	80.67 ± 0.63	82.63 ± 0.90	83.54 ± 0.35	84.70 ± 0.50	85.67 ± 0.81	86.13 ± 0.16	85.42 ± 0.30
CFCNN [17]	84.67 ± 0.62	85.63 ± 1.31	86.54 ± 0.33	87.70 ± 0.51	88.67 ± 0.62	89.33 ± 0.17	88.35 ± 0.31
1D_2DIFCNN [16]	85.67 ± 0.61	86.63 ± 1.32	87.54 ± 0.59	88.70 ± 0.52	89.67 ± 0.63	90.14 ± 0.18	89.27 ± 0.32
DG-FuseNet	94.96 ± 0.25	95.16 ± 0.22	96.60 ± 0.29	96.99 ± 0.21	99.17 ± 0.28	99.32 ± 0.15	99.76 ± 0.10

**Table 7:** Diagnostic results of HIT datasets under different signal-to-noise ratios (%).

Symbol	−10 dB	−8 dB	−6 dB	−4 dB	−2 dB	0 dB	2 dB
ATCATN [22]	42.86 ± 1.53	43.78 ± 1.60	45.13 ± 3.62	45.65 ± 2.44	46.96 ± 3.50	47.93 ± 1.56	48.95 ± 1.63
DNOCNet [19]	47.79 ± 1.54	48.59 ± 1.61	50.02 ± 3.63	52.56 ± 2.45	57.38 ± 3.51	60.95 ± 1.57	62.75 ± 1.64
PGCNN [20]	52.92 ± 3.93	55.58 ± 2.22	59.09 ± 5.21	62.08 ± 1.21	65.06 ± 0.79	66.51 ± 0.53	68.57 ± 0.85
DRCNN [21]	71.86 ± 1.47	74.05 ± 2.22	75.64 ± 2.81	78.02 ± 3.80	80.51 ± 2.63	81.86 ± 0.82	82.52 ± 0.43
DGE-GNN [23]	76.03 ± 1.67	77.05 ± 1.35	78.01 ± 1.16	79.04 ± 1.36	80.71 ± 1.59	81.66 ± 1.18	81.01 ± 1.48
ACEL [18]	80.22 ± 3.34	81.13 ± 3.99	82.41 ± 4.31	83.48 ± 0.83	83.97 ± 0.84	85.39 ± 0.97	86.17 ± 0.73
AASRN [24]	78.80 ± 2.54	79.38 ± 0.78	80.43 ± 1.21	81.42 ± 0.83	82.46 ± 0.85	82.32 ± 0.34	82.01 ± 0.65
QNN-BiLSTM [25]	71.38 ± 0.87	74.41 ± 1.51	76.58 ± 0.22	77.91 ± 0.74	79.03 ± 0.53	79.98 ± 0.40	79.95 ± 0.54
CDAE-SNN [26]	76.64 ± 0.67	78.50 ± 1.39	79.36 ± 0.75	80.47 ± 0.54	81.39 ± 0.85	81.82 ± 0.19	80.91 ± 0.33
CFCNN [17]	80.44 ± 0.69	81.35 ± 1.42	82.21 ± 0.77	83.32 ± 0.55	84.24 ± 0.87	84.67 ± 0.20	83.76 ± 0.34
1D_2DIFCNN [16]	81.39 ± 0.70	82.30 ± 1.43	83.16 ± 0.78	84.27 ± 0.56	85.19 ± 0.88	85.62 ± 0.21	84.71 ± 0.35
DG-FuseNet	93.06 ± 0.28	93.25 ± 0.25	93.67 ± 0.30	94.04 ± 0.23	94.21 ± 0.31	94.35 ± 0.16	94.63 ± 0.13

**Table 8:** Ablation variants used to evaluate the contribution of each component.

Variant	WTConvNext	FFC	DGF	Fusion Strategy	Purpose
Baseline-CNN	×	×	×	—	Verify the baseline of ordinary convolution
FFC-only	×	√	×	—	Verify the global frequency domain modeling capability
WTConvNext-only	√	×	×	—	Verify the multi-scale wavelet modeling capability
FFC + WTConvNext + Add	√	√	×	Addition	Verify the effect of simple fusion
FFC + WTConvNext + Concat	√	√	×	Concatenation	Verify static splicing fusion
DG-FuseNet	√	√	√	DGF	Verify the complete model

**Table 9:** Ablation study of different components in DG-FuseNet on the MTRG and HIT datasets.

Variant	MTRG Acc. (%)	HIT Acc. (%)	MTRG Mean Acc. Under Noise (%)	HIT Mean Acc. Under Noise (%)	Params (M)	FLOPs (G)
Baseline-CNN	47.71± 0.56	41.20 ± 1.12	40.21 ± 1.02	39.14 ± 1.12	0.92	0.31
FFC-only	76.30± 0.35	74.31 ± 0.61	73.67 ± 0.52	71.65 ± 0.69	1.18	0.39
WTConvNext-only	87.81± 0.32	83.00 ± 0.45	82.28 ± 0.35	80.52 ± 0.63	1.24	0.43
FFC + WTConvNext + Add	94.82 ± 0.21	92.77 ± 0.31	91.12 ± 0.18	90.71 ± 0.44	1.52	0.52
FFC + WTConvNext + Concat	95.12 ± 0.18	93.24 ± 0.29	92.21 ± 0.26	91.32 ± 0.40	1.67	0.57
DG-FuseNet	99.76 ± 0.10	94.32 ± 0.15	97.42 ± 0.13	93.89 ± 0.24	1.74	0.60

## Data Availability

The data featured in this study can be obtained upon request from the corresponding author. Please note that all requests for data must be made in writing and should include a detailed explanation of the intended use of the data. Upon receipt of a valid request, the corresponding author will provide the data in a timely manner. It is important to ensure that the data is used only for the purposes outlined in the request and in accordance with any applicable ethical guidelines and regulations.

## Abbreviations

The following abbreviations are used in this manuscript:

DG-FuseNet	Dual-Scale Dynamic Gated Fusion Network
WTConvNext	Wavelet Transform ConvNext Branch
MTRG	Metro Train Running Gear
HIT	Harbin Institute of Technology
SNR	signal-to-noise ratio
HOITR	High-Order Interpolation Tracking Resampling
FFC	Fast Fourier convolution
DGF	Dynamic Gated Fusion
DWT	Discrete Wavelet Transform
IWT	Inverse Wavelet Transform
FFT	Fast Fourier Transform
IFFT	Inverse Fast Fourier Transform
LP	Low-Pressure Rotor
HP	High-Pressure Rotor
CNN	Convolutional Neural Network
t-SNE	t-Distributed Stochastic Neighbor Embedding
FAG	bearing brand of Schaeffler
SKF	Svenska Kullagerfabriken
CRRC	CRRC Corporation Limited
